# Anti-angiogenic therapy using the multi-tyrosine kinase inhibitor Regorafenib enhances tumor progression in a transgenic mouse model of ß-cell carcinogenesis

**DOI:** 10.1038/s41416-023-02389-6

**Published:** 2023-08-24

**Authors:** Maren Juliane Egidi, Sebastian Krug, Johannes Haybaeck, Patrick Michl, Heidi Griesmann

**Affiliations:** 1 Clinic for Internal Medicine I, Martin-Luther University Halle/Wittenberg, Ernst-Grube-Straße 40, D 06120 Halle, Germany; 2 Department of Internal Medicine IV, Heidelberg University Hospital, Heidelberg, Germany; 3 Department of Pathology, Neuropathology, and Molecular Pathology, Medical University of Innsbruck, Innsbruck, Austria; 4 Diagnostic & Research Center for Molecular Biomedicine, Institute of Pathology, Medical University of Graz, Graz, Austria

**Keywords:** Tumour angiogenesis, Target validation, Pancreatic cancer

## Abstract

**Background:**

Pancreatic neuroendocrine tumors (PNETs) represent a distinct hypervascularized tumor entity, often diagnosed at metastatic stage. Therapeutic efficacy of anti-angiogenic multi-kinase inhibitors is frequently limited by primary or acquired resistance in vivo. This study aimed to characterize the molecular mode of action as well as resistance mechanisms to the anti-angiogenic multi-tyrosine kinase inhibitor (TKI) Regorafenib in vitro and in vivo.

**Methods:**

In vitro, human and murine pancreatic neuroendocrine cell lines were comparatively treated with Regorafenib and other TKIs clinically used in PNETs. Effects on cell viability and proliferation were analyzed. In vivo, transgenic RIP1Tag2 mice were treated with Regorafenib at two different time periods during carcinogenesis and its impact on angiogenesis and tumor progression was evaluated.

**Results:**

Compared to the established TKI therapies with Sunitinib and Everolimus, Regorafenib showed the strongest effects on cell viability and proliferation in vitro, but was unable to induce apoptosis. Unexpectedly and in contrast to these in vitro findings, Regorafenib enhanced proliferation during early tumor development in RIP1Tag2 mice and had no significant effect in late tumor progression. In addition, invasiveness was increased at both time points. Mechanistically, we could identify an upregulation of the pro-survival protein Bcl-2, the induction of the COX2-PGE2-pathway as well as the infiltration of CSF1R positive immune cells into the tumors as potential resistance mechanisms following Regorafenib treatment.

**Discussion:**

Our data identify important tumor cell-autonomous and stroma-dependent mechanisms of resistance to antiangiogenic therapies.

## Introduction

Pancreatic neuroendocrine tumors (PNETs) represent a rare hypervascular group of tumors arising within the endocrine compartment of the pancreas. This heterogeneous entity of neuroendocrine neoplasms accounts for only 1–2% of all pancreatic neoplasias. However, its incidence has rapidly increased over the last decades [1, 2]. PNETs show a highly variable biological behavior ranging from benign to highly aggressive tumors with different response rates to conventional and targeted therapies. Functioning PNETs include insulinomas, the most common PNET subtype. Patients with insulinoma become symptomatic due to specific hormonal hypersecretion syndromes and are therefore diagnosed at earlier stages compared to non-functioning tumors. The latter comprise the largest group of PNETs which have already metastasized in 60% of the cases at the time of diagnosis [3].

PNETs are classified according to the World Health Organization (WHO) tumor grading system. Based on the expression of nuclear Ki-67 antigen, G1- (0–2%), G2- (>2–20%) and G3-tumors (>20%) can be distinguished. While Grade 1 and 2 tumors are well differentiated, Grade 3 tumors are commonly poorly differentiated and therefore classified as neuroendocrine carcinomas (NEC) [4]. In addition to these tumor-autonomous properties, an increasing number of studies has demonstrated a correlation between tumor stroma characteristics and tumor progression, in particular between the infiltration of tumor-associated macrophages (TAM) and increased tumor growth and metastasis formation [5–7].

In addition to surgical resection, systemic chemotherapy and somatostatin-analogs, molecular targeted therapies have become an established treatment option for PNETs. Among them, the anti-angiogenic multi-kinase inhibitor Sunitinib and the mTOR-inhibitor Everolimus have been approved for clinical use. For both compounds, significant improvement of progression-free survival has been demonstrated. However, during the course of the disease, resistance to both drugs frequently occurs [8–10].

The multi-kinase inhibitor Regorafenib represents another promising anti-angiogenic compound. Regorafenib potently blocks several protein kinases that are involved in tumor angiogenesis (VEGFRs 1–3), oncogenesis (BRAF, RAF, RET, KIT), metastasis (FGFR, PDGFR, VEGFR3) and tumor microenvironment (TME) signaling (Tie2, CSF1R). Regorafenib was approved by the FDA for the treatment of refractory metastatic colorectal cancer and hepatocellular carcinoma, but has not been evaluated for the treatment of PNETs yet [11]. Since 2016, a phase II study is ongoing to investigate the effect of regorafenib in patients with metastatic neuroendocrine tumors (NCT02259725).

In this study, we investigated the effect of Regorafenib in human and murine pancreatic cell lines in vitro as well as in the transgenic RIP1Tag2 mouse model of ß-cell-carcinogenesis. As expected, we could confirm a pronounced anti-proliferative action of Regorafenib in vitro. Unexpectedly, however, we observed pro-proliferative and pro-invasive effects in vivo, possibly due to cues from the TME which abolished therapeutic efficacy.

## Methods

### Cell culture

In this study two human pancreatic neuroendocrine cell lines, BON-1 and QGP-1, that are frequently used models in pancreatic neuroendocrine tumor (PNET) research, were provided from Philipps-University of Marburg. The BON-1 cells derived from Pancreatic serotonin-producing neuroendocrine tumor originating from a lymph node metastasis. QGP-1 cells are derived from Pancreatic somatostatinoma. BON-1 and QGP-1 display high expression of genes associated with immature or non-functional β/δ-cell genes. BON-1 cells were cultured in DMEM F-12 (Gibco, Invitrogen Corp.) supplemented with 10% FCS (Capricorn Scientific, Ebsdorfergrund, Germany). QGP-1 cells were grown in RPMI (Gibco, Invitrogen Corp.) supplemented with 10% FCS. For further in vitro experiments BON-1 cells were seeded at a density of 7 × 10^3^ cells/96-well and 3 × 10^5^ cells/6-well. QGP-1 cells were seeded at a density of 1 × 10^4^ cells/ 96-well and 5 × 10^5^ cells/6-well.

### Inhibitors

Regorafenib (S1178), Sunitinib malate (AXON 1398) and Everolimus (Y-10218) were purchased from Selleck Chemicals, Axon medchem and MedChemExpress, respectively. The suitable concentrations were previously determined in a viability assay using a concentration series based on the literature. For the following experiments, one concentration was selected in each case by which an approximately 50% inhibition of cell viability (IC50 value) could be achieved. For in vitro studies stock concentrations were prepared in DMSO and stored according to the manufacturer’s protocol. All cell lines were treated with Regorafenib, Sunitinib and Everolimus, respectively, for 24 h and 48 h.

### Isolation and culture of primary murine bone marrow macrophages

Murine bone marrow macrophages were isolated from femur and tibia of 8-week old C57BL/6N mice. Isolated monocytes were differentiated to macrophages in RPMI supplemented with 20 ng/ml mMCSF (*macrophage stimulating colony factor*; BioLegend), 5% FCS and 1% penicillin/streptavidin (Thermo Fisher Scientific) for 7 days. For analysis macrophages were cultured in RPMI (Gibco, Invitrogen Corp.) supplemented with 5% FCS, 5 ng/ml mMCSF and were seeded at a density of 2 × 10^4^ cells/96-well and 1 × 10^5^ cells/6-well for further experiments. For polarization into M1—and M2 phenotype macrophages were incubated for 4 h with LPS (*Lipopolysaccharide*) and IFNy *(Interferon gamma*) (each 10 ng/ml; Peprotech) or IL-4 (10 ng/ml; Peprotech). Afterwards 0.5 µM Regorafenib was added into the culture media for additional 24 h.

### Isolation and culture of murine pancreatic β-tumor cell lines

Murine pancreatic β-tumor cell lines were established from 15 week old RIP1Tag2-mice (B6.D2-Tg(RIP1Tag2) 2Dh) as described by Efrat et al. [12]. For generating the cell lines insulinoma were dissected from the pancreata, disrupted and transferred to cell culture dishes with DMEM media containing 2.5% FCS and 15% horse serum (Gibco, Invitrogen Corp.). Three murine pancreatic β-tumor cell lines (HMEG 1-3) were cultured for further analysis in DMEM supplemented with 10% FCS. HMEG cells show a different mutation pattern compared to the human cells. HMEG and QGP-1 proliferate significantly slower than the BON-1 cells. For further experiments HMEG cells were seeded at a density of 4 × 10^4^ cells/96-well and 8 × 10^5^ cells/6-well. These cells were not used for comparison with the human cells, but rather to make predictions for the response to Regorafenib in the mouse model afterwards.

### Treatment of insulinoma ex vivo

To remove insulinomas from the pancreas of 15-week old RIP1Tag2 mice, the mice were sacrificed by cervical dislocation. Subsequently, the collagenase P solution (1.5 mg/ml; Sigma-Aldrich) was injected into the exocrine pancreas via the bile duct of the mice. After removal the pancreas was placed in a 50 ml centrifuge tube with 3 ml of Collagenase P solution and shaken for 10 min at 37 °C. This was followed by a threefold vigorous vertical shaking by hand, and the insulinomas could be separated from the exocrine part of the pancreas. The isolated insulinomas were then subdivided into two groups based on the size (small: 2 mm in diameter, large: 3–4 mm in diameter) and the angiogenic status (weakly angiogenic: light red, highly angiogenic: dark red).

The prepared insulinomas were then cultured in 1.5% agarose-coated 12-well plates in RPMI medium with 10% FCS. The next day, the insulinomas were treated with 10 μM Regorafenib for 48 h followed by protein analysis.

### Cell viability

Cell viability was measured using *CellTiter-Glo Luminescent Cell Viability Assay* (Promega). Cells were seeded in a volume of 100 µl in 96-well plates and were treated with Regorafenib, Sunitinib and Everolimus (for concentrations see figures) for 24 h and 48 h. To analyze the effect of PGE2 (Prostaglandin E2) cells were pretreated with 10 µM Regorafenib for 2 h and 10 µM of PGE2 (Selleckchem, S3003) was added for further 22 h. 100 µl of *CellTiter-Glo*®-reagent was added to every well and luminescence was measured using the Luminoskan Ascent (Thermo Scientific).

### Proliferation assays

BON-1 and QGP-1 cells were seeded in 24-well culture plates and treated with 6 µM Regorafenib, 10 µM Sunitinib and 1 µM Everolimus respectively for 24 h and 48 h. HMEG cells were seeded in 12-well culture plates and were treated with 10 µM Regorafenib for 24 h and 48 h. Cells were harvested and absolute cell count was determined.

### Bioenergetic analyses

After Regorafenib, Sunitinib and Everolimus treatment, respectively, the key parameters of mitochondrial function oxygen consumption rate (OCR) and extracellular acidification rate (ECAR) were analyzed using the Seahorse XF Cell Mito Stress Test Kit by the XF96 extracellular flux analyzer (Agilent Technologies) as previously described. Briefly, cells were seeded in triplicates in XF96 Cell Culture Microplates and treated with the respective inhibitor for 24 h. The cell medium was changed into unbuffered, serum-free DMEM (Seahorse) supplemented with 10 mM glucose, 1 mM pyruvate, and 2 mM glutamine. The OCR and ECAR were measured in a time course after injection of oligomycin (2 µM), FCCP (1 µM) and rotenone/antimycin (0.5 µM).

### Flow cytometry analysis

For cell cycle analysis cells were seeded in six-well-culture plates and treated with Regorafenib, Sunitinib and Everolimus respectively for 24 h and 48 h. After incubation, cells were harvested, fixed in ice cold 70% ethanol and kept at 4 °C for 7 days. After that cells were washed two times, re-suspended in DNA staining solution (PBS with 10 µg/ml Propidium iodide and 100 µg/ml RNase A) and after 30 min incubation at 37 °C in the dark DNA content was measured.

The effect of Regorafenib on HMEG cells after 48 h was analyzed by the Click-iT™ EdU Alexa Fluor™ 488 Imaging Kit (Thermo Fisher Scientific) according to the manufacturer’s protocol. The amount of fluorescence signal corresponding to the intercalating EdU (5-Ethynyl-2′-Deoxyuridin) into the DNA was measured. All flow cytometry measurements were done with the LSR II FortessaTM (BD Biosciences Systems) and all data were analyzed using the FlowJo software.

### Western blotting

Cells were harvested, centrifuged and the pellet was resuspended in RIPA-buffer supplemented with protease and phosphatase inhibitor. Cells were sonicated and protein content was measured using Coomassie Plus Bradford Assay Kit (Thermo Scientific). Proteins were electrophoresed on SDS-polyacrylamide gels and transferred to PVDF membranes. Membranes were blocked in 5% nonfat dry milk or 5% BSA (*Bovine serum albumin*) in TBS-T (0.1% Tween-20) for 1 h and were incubated with appropriated primary antibody over night at 4 °C, followed by an incubation of secondary antibody for 1 h according to the manufacturer’s protocol. The following antibodies were used: Bcl-2 (#7382); CSFR (#692); PCNA (#56) were obtained from Santa Cruz; Cyclin D3 (#2936), PARP-1 (#9542); S6 Ribosomal protein (#2217); Phospho-S6 Ribosomoal protein (Ser235/236) (#2211) from Cell Signaling; pCSF1R (Tyr723) (#MA5-15151) from Thermo Fisher Scientific; ß-actin (#A1978) from Sigma; PTGES2 (#OAAN02154) from AvivaSysbio. ECL Mouse IgG, HRP-linked (#NA931), ECL Rabbit IgG, HRP-linked (#NA934) from GE Healthcare. The INTAS LabImage 1D software was used for quantification of protein expression normalized to ß-actin and albumin, respectively.

### qPCR analysis

RNA from HMEG cell lines was isolated using the NucleoSpin® RNA Kit from Macherey & Nagel. RNA from paraffin embedded tissue was isolated using the RNeasy FFPE Kit from Qiagen Kit. RNA isolations were according to the manufacturer’s protocol. cDNA was synthesized from 1 µg RNA using random primers and Omniscript Reverse Transcription (RT) Kit (Qiagen). Quantitative real-time reverse transcription PCR was performed using SYBR Green Master Mix (NEB) on a 7500 Fast Realtime PCR system (Applied Biosystems). The ribosomal protein RPLP0 was used as housekeeping gene. All experiments were performed in duplicates and are displayed in ±SD. The primers were supplied by Biomers. The sequences are provided in Supplementary Table 1.

### Animal models and treatment scheme

The generation and characterization of the transgenic RIP1Tag2 mouse model (B6.D2-Tg(RIP1Tag2)) has been described previously [13, 14]. Mice were purchased from NCI Mouse Repository and kept on a C57BL/6N background. No glucose-enriched food or water was given during experimental studies. Male and female mice were treated from week 6–9 (during tumor development; *n* = 13) or week 9–15 (during tumor progression; *n* = 17) with 10 mg/kg bw/day Regorafenib solved in a volume of 100 µl polyethylenglycol (PEG)/methane sulfonic acid (80:20) per oral gavage 5 days a week followed by a 2-day break. 100 µl of solvent solution was used for control groups. State of health and body weight of the mice was documented before application. After treatment for 4 weeks (tumor development) and 6 weeks (tumor progression) mice were sacrificed. Blood and tissues were obtained and processed for Western blot, histology and PCR analysis. Blood recovered from the pericardium was allowed to clod on ice and serum was collected by centrifugation at 10.000 rpm for 5 min. Aliquots were stored at −80 °C. All animal experiments were approved by the local government authorities and performed according to the guidelines of the animal welfare committee. No animals were excluded from final analysis. At least six mice per treatment group were used to reach statistical significance, blinding of the treatment groups to the researchers was not feasible. The primer sequences used for genotyping of RIP1Tag2 mice by classical PCR were supplied by Biomers. The sequences are provided in Supplementary Table 1.

### Histology and immunohistochemistry

Dissected pancreata were fixed in 3.7% PFA overnight. Organs were dehydrated in graded ethanol series and embedded in paraffin. Paraffin sections were stained with hematoxylin and eosin (H&E) following standard procedures. Immunohistochemical stainings were performed using the Dako Envision AEC Kit (#K4009, Dako, Germany) for antibody detection according to the manufacturer’s instructions. Anti-Bcl-2 (Epitomics; #EP36), Anti-Cathepsin B (Bioss; # bs-1500), Anti-CD3 (Thermo Scientific; # MA1-39551), Anti-CD31 (Dianova; # DIA-310), Anti-CSF1R (Abcam; #215441), Anti-F4/80 (NOVUS; #NB600-404), Anti-Ki-67 (DAKO; # M7249) were incubated 1 h at room temperature. Immunohistochemical stainings and assessment as well as tumor grading were performed by two independent assessors (M.J.E., J.H.). In addition, slides were scannend with the AxioScan Z.1 (Zeiss) and analyzed by StrataQuest Analysis Software (TissueGnostics) using specific generated templates. These templates enable information about the size of the whole tissue, the discrimination between tumor and healthy tissue as well as the amount of DAB-staining.

Each insulinoma was classified using the grading system (NET G1-G3 and NEC G3) according to WHO standards (2017) for neuroendocrine tumors. This classification most accurately addresses differences in proliferation influenced by Regorafenib and is clinically used to stage neuroendocrine tumors. This grading is based on the proliferation rate of the tumors using Ki-67 as a marker for cell division rate. After determining the expression of nuclear Ki-67 antigen in percent of positive nuclei, tumors are classified in G1- (0–2%), G2- (>2–20%) and G3-tumors (>20%). While grade 1 and 2 tumors are well differentiated, tumors with higher proliferation rates are less well differentiated and therefore classified as G3 neuroendocrine tumors (G3 NET) or NEC. To address the differences between G3 NET and NEC, highly proliferating tumors were further grouped into tumors showing a Ki-67-index of 20–55% (corresponding to G3 NET) and higher than 55% (resembling NEC), respectively. This classification is also being used in patients to identify NEC with highly aggressive behavior.

In addition, each islet was grouped into non-invasive and invasive insulinoma regarding to the quality of the insulinoma capsule. While tumors with smooth tumor border (adenoma) were classified as non-invasive, tumors with no more than 1 to 2 microinvasions (IC1) or macroinvasive carcinomas (IC2) were classified as invasive tumors. The lesions were classified in two groups (non-invasive vs. invasive), with the invasive lesions consisting of both IC1 and IC2 lesions.

### Statistical analysis

Data of in vitro experiments are representative for at least three independent experiments and are presented as means ± SD. Statistical evaluation was performed by the use of a two-tailed unpaired Student’s *t* test. The statistical significance of the pancreata from RIP1Tag2 mice was done by two-side Mann-Whitney *U* Test. Survival curves were calculated using the Kaplan-Meier method. Log-rank test was applied to identify significant differences. Significance of the amount of invasive islets and grading was performed by Chi-square test. All statistical analysis was performed by Graph Pad Prism 5 Software.

The following nomenclature was used to indicate the significance: ns = non-significant;

*p = 0.01–5: significance; **p = 0.01–0.001: medium significance ***p < 0.001: high significance.

## Results

### Regorafenib reduces cell proliferation and viability in vitro

The multi-kinase inhibitor Regorafenib has been frequently used in several in vitro and mouse xenograft models with a proven effect on cell proliferation and cell viability [11, 15, 16]. To study the impact of Regorafenib in PNET cells in vitro, we used two human neuroendocrine cell lines (BON-1, QGP-1) and three murine pancreatic β-tumor cell lines (HMEG1-3) derived from 15-week-old RIP1Tag2 mice.

**Fig. 1:**
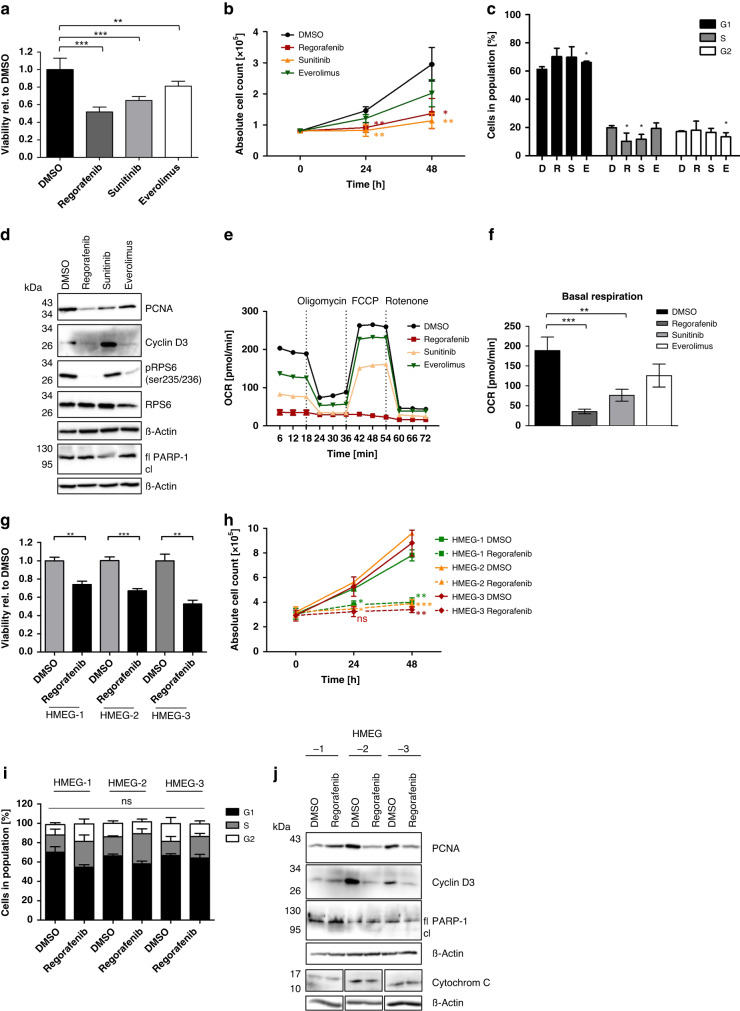
Regorafenib reduces cell viability and cell proliferation in BON-1 cells and murine pancreatic β-tumor cells without induction of apoptosis. BON-1 cells were treated with 6 µM Regorafenib, 10 µM Sunitinib, 1uM Everolimus or 0.1% DMSO for 24 h and 48 h, respectively (**a**–**f**). **a** Effect on cell viability was determined by ATP-based CellTiter Glo Assay after 48 h. Values are shown relative to DMSO-treated control (*n* = 3). **b** Cell proliferation shown as absolute cell counts after 24 h and 48 h (*n* = 3). Statistical significance of respective treatment compared to control after 24 h and 48 h respectively. **c** Cell cycle analysis by flow cytometry using Propidium iodide depicting percentage of cells in G1, S-, and G2-phase after 48 h treatment with DMSO (D), Regorafenib (R), Sunitinib (S) and Everolimus (E), respectively. Unless otherwise stated, the *p* values determined from the cell cycle analysis are not significant (*n* = 3). **d** Representative Western blot analysis showing expression of proliferation and translation markers as well as apoptotic protein PARP-1 (fl: full-length PARP-1; cl:cleaved PARP-1 fragment at 89 kDa). β-actin was used as loading control. Mitochondrial respiration after 24 h treatment depicted in a temporal course (**e**) and bar chart (**f**). Oxygen consumption rate (OCR) in pmol/min. Recordings took place under basal, oligomycin-inhibited, FCCP-induced maximal and rotenone + antimycin A-inhibited (rotenone) conditions. Murine pancreatic β-tumor cells were treated with 10 µM Regorafenib or 0.1% DMSO for 24 h and 48 h, respectively (**g**–**j**). **g** Effect on cell viability after 48 h was assessed by an ATP-based CellTiter Glo Assay. Values are shown relative to DMSO-control (*n* = 3). **h** Cell proliferation was assessed as absolute cell count after 24 h and 48 h (*n* = 3). Statistical significance of Regorafenib treatment compared to control after 24 h and 48 h respectively. **i** Cell cycle analysis was performed by flow cytometry using Propidiumiodid staining, determined as percentage of cells in G1-, S- and G2-phase (*n* = 3). Statistical significance could not be reached. **j** Representative Western blot analysis showing expression of proliferation marker PCNA and Cyclin D3 (*n* = 3) as well as Cytochrom C, Bcl-2 (*n* = 2), Bcl-XL, cleaved caspase 3 (*n* = 2) and apoptotic protein PARP-1 (fl: full-length PARP-1; cl: cleaved PARP-1 fragment at 89 kDa) (*n* = 3). β-actin was used as a loading control. All protein lysates were run on one single gel, but additional treatment conditions that were originally loaded on the gel were cut out. The uncropped blots are attached in the supplementary materials section. Statistical evaluation was performed by the use of a two-tailed unpaired Student’s *t* test. *p ≤ 0.05; **p < 0.01; ***p < 0.001 ns = non-significant.

First, we compared the in vitro effects of Regorafenib with the two established targeted therapies in PNETs, the anti-angiogenic multikinase inhibitor Sunitinib and the mTOR inhibitor Everolimus (Fig. 1a–f for BON-1 cells; Supplementary Fig. S1 for QGP-1 cells). Treatment of BON-1 cells with Regorafenib (6 µM), Sunitinib (10 µM) and Everolimus (1 µM), respectively, reduced both the metabolic activity determined by impaired ATP-production (Fig. 1a) and the proliferation rate (Fig. 1b) both after 24 or 48 h (Supplementary Fig. S2A+B). Reduced proliferation correlated with reduced S-phase progression and G1-phase arrest, as measured by flow cytometry analysis (Fig. 1c). All effects on proliferation and cell cycle progression were most pronounced upon Regorafenib treatment, followed by Sunitinib, and were least pronounced after Everolimus treatment.

On protein level, we could confirm the impact of Regorafenib on cell proliferation in BON-1 cells by a marked reduction in the proliferation markers PCNA (Proliferating-Cell-Nuclear-Antigen) and Cyclin D3 as well as of the translation associated protein RPS6 (protein ribosomal protein S6). In comparison, Sunitinib predominantly affected proliferation while Everolimus reduced protein translation only. Notably, neither inhibitor was able to induce apoptosis to a significant extent, as measured by PARP-1 cleavage (Fig. 1d).

Since inhibition of the respiratory chain with a consecutively reduced supply of ATP could affect anti-proliferative effects, we investigated the impact of the inhibitors on cell metabolism.

Metabolic analysis using the Seahorse analyzer showed a reduction of OCR for all three inhibitors after 24 h. However, only Regorafenib caused a complete uncoupling of the mitochondrial respiratory chain (Fig. 1e+f). Similar but less pronounced effects on OCR were detected even after short-course treatment with Regorafenib for 1 h (Supplementary Fig. S3A+B). This indicates a specific and time-dependent effect on mitochondria which is not secondary to reduced cell proliferation. Anaerobic glycolysis (ECAR; *extracellular acidification rate*) was also slightly affected by all three inhibitors (Supplementary Fig. S3C+D).

Prior to evaluating the effects of Regorafenib in vivo using the RIP1Tag2 mouse model, we aimed to corroborate our findings observed with human PNET cell lines in appropriate murine cell lines. Therefore, we established and propagated three murine pancreatic β-tumor cell lines termed HMEG1-3, derived from 15-week-old RIP1Tag2 mice.

First, we confirmed that all HMEG cell lines showed all characteristics of pancreatic neuroendocrine cells by verifying their strong expression of SV40 large T antigen, PDX1 (*Pancreas/duodenum homeobox protein 1*) and chromogranin A on protein and insulin on RNA level (Supplementary Fig. S4A+B).

Similar to the results observed in human cell lines, Regorafenib reduced the metabolic activity due to impaired ATP-production in all three HMEG cell lines (Fig. 1g) time- and dose-dependently (Supplementary Fig. S5A). In addition, cell counting revealed a complete proliferation arrest (Fig. 1h). In contrast to the human cell lines, cell cycle analysis did not show the G1 arrest detected in the human cell lines (Fig. 1i). We subsequently used the EdU-Click flow cytometry method in which the thymidine analog EdU (5-ethynyl-2′-deoxyuridine) is incorporated only into newly synthesized DNA and proliferating cells can be distinguished from resting cells based on the observed fluorescence signal. These data showed that the effect on cell cycle is most likely due to an accumulation of cells in S-phase without entering the G2-phase (Supplementary Fig. S5B+C).

On protein level, we confirmed the inhibitory effect of Regorafenib on cell proliferation by detecting strongly reduced protein expression of PCNA and Cyclin D3. As observed in the human cell lines, Regorafenib-treated HMEG cell lysates showed no signs of apoptosis, indicated by absent PARP-1 and caspase 3 cleavage as well as cytochrome C release. Furthermore, no differences in protein expression of Bcl-2 and Bcl-XL could be observed after Regorafenib treatment (Fig. 1j).

### Regorafenib enhances proliferation in vivo during early tumor development

**Fig. 2:**
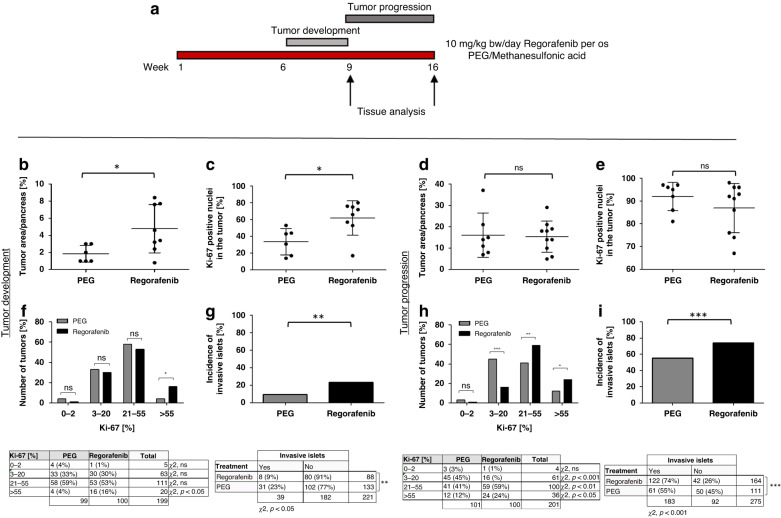
Regorafenib leads to a higher proliferation rate during early tumor development without significant effect during late tumor progression. **a** Treatment scheme of oral application of Regorafenib (10 mg/kg bw/day) or PEG control solution from week 6–9 (during tumor development) or week 9–15 (during tumor progression) in the transgenic RIP1Tag2-mouse model. **b** + **d** Percentage of the tumor area relative to the total area determined in longitudinal pancreas sections, **c** + **e** percentage of Ki-67-positive nuclei of Regorafenib- and PEG-treated mice during tumor development (PEG: *n* = 6; Rego: *n* = 8) and tumor progression (PEG: *n* = 7; Rego: *n* = 10). *p ≤ 0.05; ns not significant by Mann–Whitney *U* test. **f** + **h** Percentage of tumors classified according to their grading according to the Ki-67-index and (**g** + **i**) percentage of invasive versus non-invasive tumors in Regorafenib- and PEG-treated mice during early tumor development and late tumor progression. Contingency table below depicts the number and ratio of graded tumors (Ki-67%-index) and invasive and non-invasive tumors in each treatment group by Fisher’s exact test *p ≤ 0.05; **p < 0.01; ***p < 0.001; ns = non-significant.

To evaluate the effect of Regorafenib in vivo, we used the RIP1Tag2 transgenic mouse model. RIP1Tag2 mice express the SV40 large T antigen under the control of the rat insulin promoter, leading to a stepwise progression from normal to angiogenic, hyperplastic and invasive islets [13, 14]. We treated the mice from week 6 until 9 (during early tumor development) or week 9 until 15 (during tumor progression) with 10 mg/kg bw/day for 5 days followed by a 2-day-break (Fig. 2a).

During early tumor development, we observed an unexpected increase in tumor size following Regorafenib treatment (1.8% vs. 4.8% of the total pancreas area; *p* = 0.04) (Fig. 2b), accompanied by a significantly increased Ki-67 positivity index with 62% vs. 33% positive nuclei in Regorafenib- compared to PEG-treated control mice (*p* = 0.02) (Fig. 2c; see Ki-67 staining of the total pancreas in Supplementary Fig. S6a). If Regorafenib was administered during tumor progression, no difference in tumor area and Ki-67 positivity between Regorafenib- and PEG-treated animals could be detected anymore (Fig. 2d+e; Supplementary Fig. S6C). Interestingly, we observed a significantly higher incidence of highly proliferating (Ki-67 > 55%) (Fig. 2f+h) and invasive tumors following Regorafenib during both treatment periods (Fig. 2g+i). Since mice were sacrificed at defined time points, we were unable to analyze survival differences (Supplementary Fig. S6E+F). These data indicate that Regorafenib paradoxically increases proliferation during early tumor development. At later stages, tumor size was not significantly different, but the proportion of highly proliferative and invasive tumors remained persistently higher after Regorafenib treatment.

### Regorafenib fails to inhibit tumor angiogenesis during tumor development

Angiogenesis, a hallmark of cancer, represents an important step in the development, resistance and growth of highly vascularized tumors such as PNETs. Regorafenib is known to exert its anti-angiogenic properties by blocking VEGF receptors 1–3.

**Fig. 3:**
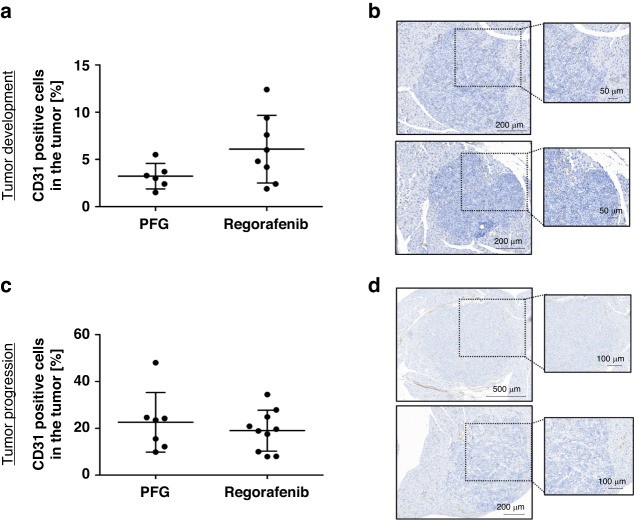
Blood vessel density within the tumors after Regorafenib treatment. **a** + **c** Percentage of CD31-positive cells in the tumors. Boxplot data are presented as mean ± SD by Mann–Whitney *U* test. No statistical difference was found. **b** + **d** Representative CD31-staining of PEG- and Regorafenib- treated mice during tumor development (PEG: *n* = 6; Rego: *n* = 8) and tumor progression (PEG: *n* = 7; Rego: *n* = 10).

Surprisingly, however, Regorafenib had no significant effects on the formation of blood vessels in the RIP1Tag2 model, as assessed by immunohistochemistry. The tumors in particular from both treatment periods, during early tumor development and later during tumor progression, showed no significant difference in CD31 positive cells following Regorafenib treatment (Fig. 3a+c; see CD31 staining of the total pancreas in Supplementary Fig. S7A+B). Representative immunhistochemical CD-31 staining’s are shown in Fig. 3b+d.

To investigate if angiogenic ligands are regulated as potential compensatory mechanism following VEGFR2 blockade, we analyzed VEGF-A levels in the sera of the mice by Western blot. Interestingly, densitometric analysis showed no significant differences in VEGF-A levels in the early treatment phase, but a clear and significant increase following Regorafenib treatment during tumor progression, indicating a compensatory upregulation of VEGF-A upon Regorafenib treatment which might contribute to its lack of efficacy on angiogenesis (Supplementary Fig. S7C–F).

### Regorafenib leads to an increase of tumor-infiltrating M2 macrophages

**Fig. 4:**
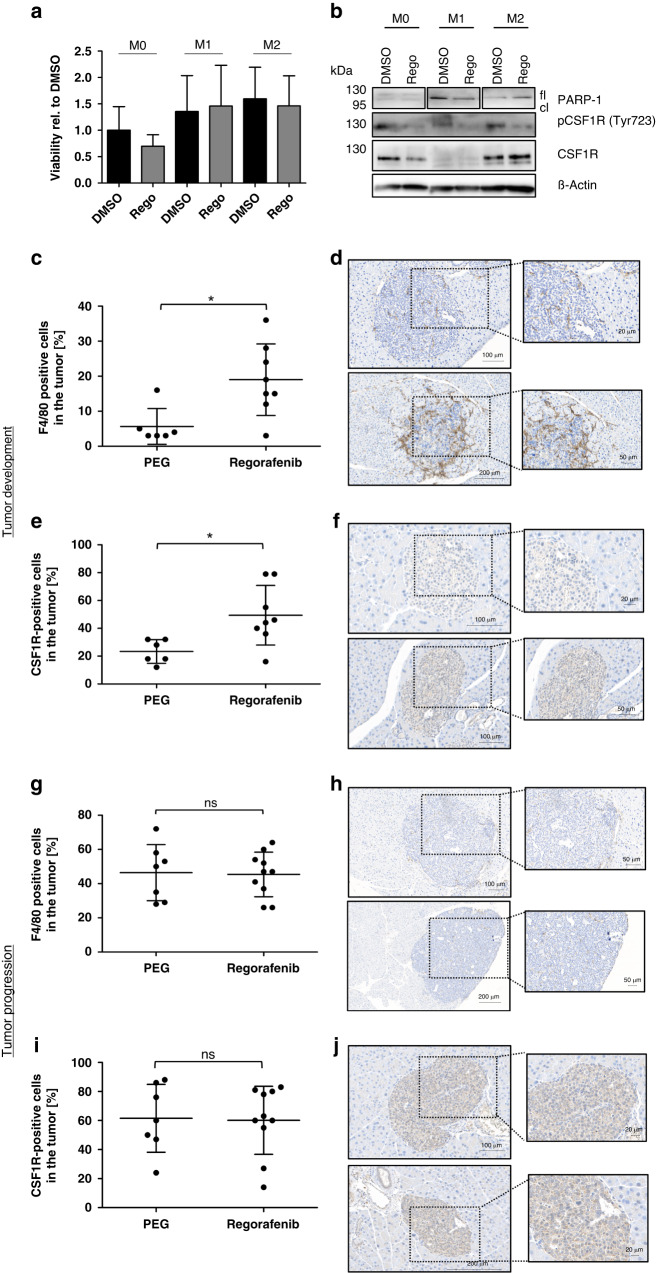
Regorafenib induces a tumor-promoting micromilieu by enhancing the infiltration of M2-macrophages. Murine bone marrow macrophages from non-transgenic mice were polarized into M1 and M2 macrophages (or left untreated) and were treated with 0.5 µM Regorafenib for 24 h. **a** Effect on cell viability was assessed by an ATP-based CellTiter Glo Assay. Values are shown relative to DMSO-control. **b** Western blot analysis showing PARP-1 (*n* = 3) as well as total (*n* = 3) and phosphorylated amount of CSF1R (Tyr723) (*n* = 2) (fl: full-length PARP-1; cl: cleaved PARP-1 fragment at 89 kDa). β-actin was used as a loading control. All protein lysates were run on one single gel, but additional treatment conditions that were originally loaded on the gel were cut out. **c** + **g** Percentage of F4/80-positive cells in the tumor, **d** + **h** representative F4/80-staining and (**e** + **i**) percentage of CSF1R-positive cells in the tumor of Regorafenib- and PEG-treated mice during tumor development (PEG: *n* = 6; Rego: *n* = 8) and tumor progression (PEG: *n* = 7; Rego: *n* = 10) with representative CSF1R-staining (**f** + **j**). Boxplot data are presented as mean ± SD by Mann–Whitney *U* test. *p ≤ 0.05; ns=non-significant.

To elucidate the paradoxical effects of Regorafenib on tumor progression and angiogenesis in vivo, we evaluated its impact on tumor-associated macrophages (TAMs). TAMs are frequently functioning as immunosuppressive, M2-polarized macrophages which are able to promote angiogenesis and tumor progression, therefore conferring therapy resistance. The stroma of PNETs is densely populated by TAMs [5]. To evaluate if Regorafenib affects TAMs, we first analyzed the susceptibility of unpolarized (M0), M1- and M2-polarized macrophages to Regorafenib treatment in vitro. To this extent, we polarized murine bone marrow-derived macrophages into M1 and M2 phenotypes in vitro by LPS/IFNγ and IL-4, respectively, and confirmed the polarization using several M1/M2 markers (Supplementary Fig. S8A+B). Interestingly, both M1 and M2 macrophages did not show any susceptibility to treatment with Regorafenib (Fig. 4a).

The colony-stimulating factor-1 receptor (CSF1R), essential for M0 macrophage viability, has been reported as a target structure of Regorafenib [5, 17, 18]. Therefore, we analyzed the CSF1R expression and macrophage activation after Regorafenib treatment.

Interestingly, CSF1R protein levels were downregulated only in M0 macrophages following Regorafenib. M1 macrophages showed only minimal CSF1R expression, and in M2 macrophages CSF1R levels remained unchanged. In contrast, Regorafenib clearly reduced phosphorylation of CSF1R in all macrophages (Fig. 4b). However, Regorafenib was not able to induce apoptosis in M1 and M2 macrophages, but led to a PARP cleavage in M0 macrophages to a lower extent (Fig. 4b).

To determine the impact of Regorafenib on tumor-associated macrophages in vivo, we evaluated the murine macrophage marker F4/80 as well as CSF1R expression in the pancreata of Regorafenib- and PEG-treated mice by immunohistochemistry. During tumor development both F4/80 and CSF1R positive cells within the tumors significantly increased after Regorafenib administration (Fig. 4c–f; see F4/80 and CSF1R staining of the total pancreas in Supplementary Fig. S9A), indicating that infiltration of macrophages with a tumor-promoting phenotype as potential compensatory mechanism conferring therapy resistance. At later stages of tumor progression, the impact of Regorafenib on macrophage infiltration was no longer detectable (Fig. 4g–j; see F4/80 and CSF1R staining of the total pancreas in Supplementary Fig. S9B). To address the potential impact of Regorafenib on the number of lymphocytes, we performed additional immunohistochemistry stainings against CD3 on murine pancreata of Regorafenib and PEG-treated RIP1Tag2 mice during tumor progression. However, the analyses did not show any significant differences in the number or distribution of CD3 positive lymphocytes (Supplementary Fig. S9C).

### Upregulation of anti-apoptotic Bcl-2 protein in vivo

Resistance to apoptosis via upregulation of pro-survival proteins of the Bcl-2-protein family is a hallmark of cancer and one of the most frequent resistance mechanisms to various therapeutic approaches [19]. In PNETs, increasing levels of Bcl-2 expression have been shown to correlate with a higher mitotic index and Ki-67 positivity [20]. Therefore, we aimed to investigate whether Bcl-2 upregulation is also linked to the increased proliferation rate detected in Regorafenib-treated RIP1Tag2 mice.

**Fig. 5:**
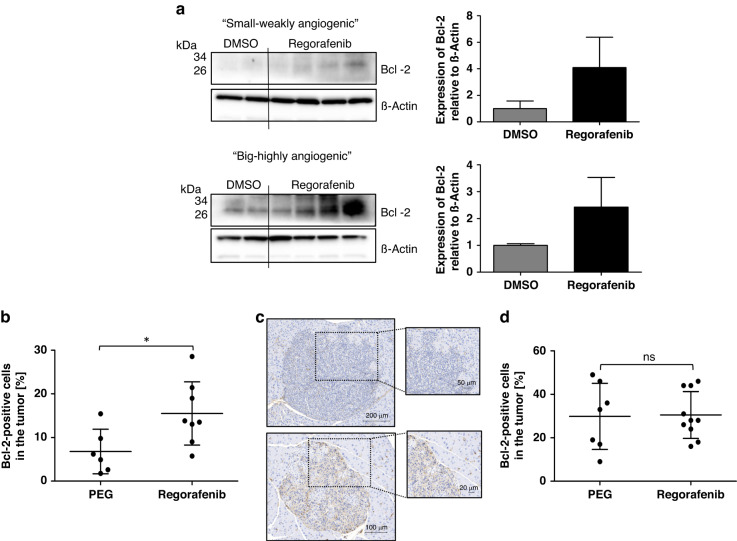
Regorafenib induces an upregulation of pro-survival Bcl-2-proteins ex vivo and in vivo. **a** Westernblot analysis of Bcl-2-expression of isolated insulinoma from RIP1Tag2 mice after treatment with 10 µM Regorafenib or DMSO for 48 h; “small-weakly angiogenic” (DMSO: *n* = 2; Rego: *n* = 4) and “large-highly angiogenic” (DMSO: *n* = 2; Rego: *n* = 4) properties with statistical analysis of Western blotting with error bars designating standard deviation of the mean. No statistical difference was found. **b** Percentage of Bcl-2-positive cells in the tumors and (**c**) representative Bcl-2-staining during tumor development (PEG: *n* = 6; Rego: *n* = 8) as well as (**d**) percentage of Bcl-2-positive cells in the tumors during tumor progression (PEG: *n* = 7; Rego: *n* = 10) of Regorafenib- and PEG treated mice. Boxplot data are presented as mean ± SD by Mann–Whitney *U* test. *p ≤ 0.05; ns = non-significant.

For this purpose, we isolated murine insulinomas from 15-week-old RIP1Tag2 mice. The islets were grouped according to size and extent of angiogenesis into two groups (“small - weakly angiogenic” and “large - highly angiogenic”) and subsequently treated with Regorafenib for 48 h. Western blot analysis indeed revealed a marked but not significant upregulation of Bcl-2 after incubation with Regorafenib in most insulinoma protein lysates compared to DMSO-treated control lysates ex vivo (Fig. 5a).

These results were confirmed on insulinoma tissues derived from mice treated during the early tumor development phase. Regorafenib treatment was associated with a significantly higher proportion of Bcl-2 positive cells in the tumors, assessed by immunohistochemistry (Fig. 5b; Supplementary Fig. S10A). After the later treatment period (tumor progression), in which no difference in tumor area and proliferation rate had been observed, the difference in Bcl-2 positivity between treatment groups was also not persisting (Fig. 5d; see Bcl-2 staining of the total pancreas in Supplementary Fig. S10B). These data indicate that Bcl-2 upregulation might represent a cellular resistance mechanism to Regorafenib during early tumor development in vivo.

### Immune cell mediated resistance mechanism via the COX2-PGE2-pathway

Since we demonstrated both an increased macrophage infiltration into the tumor and an upregulation of Bcl-2 within the tumor as potential mechanisms explaining tumor cell resistance to Regorafenib, we sought to elucidate whether both mechanisms are connected, in particular, if Bcl-2 upregulation is mediated by tumor-infiltrating macrophages.

In this context, cyclooxygenase 2 (COX2) has been shown to regulate proliferation, angiogenesis and metastasis formation in various tumors and therefore represents an important driver of carcinogenesis [21, 22]. PGE2, an enzymatic product of COX2, is overexpressed in several cancers, including pancreatic cancer cell lines. In addition to tumor cell-autonomous effects, PGE2 has been demonstrated to shift the TME towards an immunosuppressive phenotype [23, 24]. Based on these data, we aimed to study the impact of Regorafenib on the COX2-PGE2-pathway in BMM and the consequences on HMEG cells in vitro.

**Fig. 6:**
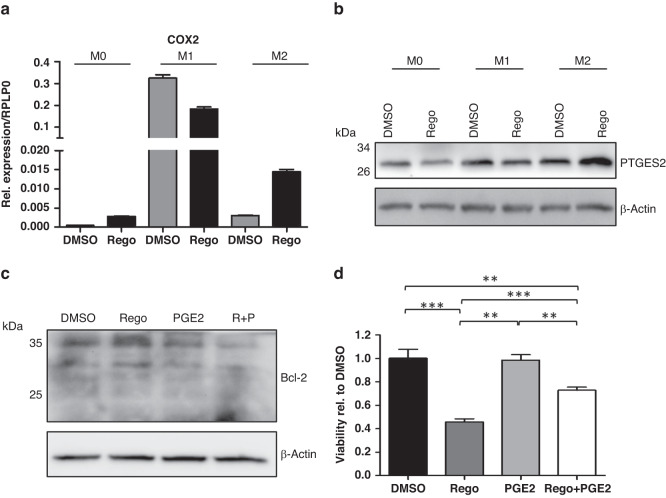
Regorafenib activates the COX2-PGE2-signaling. Murine bone marrow macrophages from non-transgenic mice were polarized into M1 and M2 macrophages (or left untreated) and treated with 0.5 µM Regorafenib for 24 h. **a** The amount of COX2 on RNA level was evaluated by qRT-PCR and normalized to RPLP0. **b** Representative Western blot analysis showing PTGES2 with statistical analysis of Western blotting pooled from two independent experiments with error bars designating standard deviation of the mean. β-actin was used as a loading control. **c** Representative Western blot analysis showing Bcl-2. β-actin was used as a loading control. Statistical significance could not be reached. **d** Murine pancreatic β-cells were pretreated with 10 µM Regorafenib for 2 h and 10 µM PGE2 was added for further 22 h. Control reaction with either Regorafenib or PGE2 alone. Effect on cell viability was assessed by an ATP-based CellTiter Glo Assay. Values are shown relative to DMSO-control. Statistical evaluation was performed by the use of a two-tailed unpaired Student’s *t* test. **p < 0.01; ***p < 0.001.

After Regorafenib treatment, COX2 mRNA was strongly upregulated in M2 macrophages and showed a slight increase in M0 macrophages without reaching significance in vitro (Fig. 6a). On protein level the increased expression of PTGES2, the synthase which is required for PGE2 synthesis, in M2-macrophages after Regorafenib treatment could further confirm the importance of the COX2-PGE2 signaling pathway (Fig. 6b).

COX2 levels have been described to correlate with secreted PGE2 levels in tumor-associated macrophages [23, 24]. To examine whether macrophage-derived PGE2 has an impact on Bcl-2 levels in the tumor cells, HMEG cells were incubated with PGE2 with or without pre-treatment with Regorafenib. As expected, Western blot analysis showed an induction of Bcl-2 after Regorafenib treatment, but neither PGE2 alone nor in combination with Regorafenib could further increase Bcl-2 levels (Fig. 6c). This indicates that Regorafenib-induced upregulation of Bcl-2 is a tumor-cell autonomous resistance mechanism which is not dependent on non-autonomous TAM-derived PGE2 secretion.

However, the viability of HMEG cells treated with PGE2 in combination with Regorafenib was significantly higher than with Regorafenib monotherapy (Fig. 6d) supporting the hypothesis that Regorafenib-induced COX2-PGE2 signaling seems to be a second independent mechanism facilitating tumor resistance and progression independently of the tumor cell-autonomous Bcl-2 upregulation.

## Discussion

In this study, we describe striking differences between in vitro efficacy of the anti-angiogenic multi-kinase inhibitor Regorafenib in pancreatic neuroendocrine cell lines and the unexpected tumor-promoting effects in vivo in a mouse model of ß-cell carcinogenesis.

As described in other cell systems, Regorafenib significantly reduced cell viability and cell proliferation in all tested human and murine pancreatic neuroendocrine cell lines.

However, in contrast to reports in other tumor cells, Regorafenib was unable to induce apoptosis in PNET cell lines [25–27]. These findings were confirmed by cell counting experiments indicating that cell numbers are not declining over 48 h after Regorafenib exposure and FACS analyses without signs of apoptosis, corroborating the hypothesis that cells undergo G1 arrest.

Notably, treatment with Regorafenib resulted in a complete uncoupling of oxidative phosphorylation (OXPHOS), whereas the two TKI’s approved in PNET´s, the antiangiogenic multi-kinase inhibitor Sunitinib and the mTOR inhibitor Everolimus, merely led to a moderately reduced OCR. Several studies reported that Regorafenib and the structurally related Sorafenib exhibited significant mitochondrial toxicity as shown in isolated mitochondria from rat liver cells. In HepG2 cells, it could be shown that Regorafenib affects the complexes II, III and IV of the mitochondrial respiratory chain [28, 29]. These reports are consistent with our data in PNET cells. In comparison to Sunitinib and Everolimus, Regorafenib has been reported to target a broader spectrum of kinases including Raf, Braf and p38 MAPK that are neither targeted by Sunitinib nor Everolimus. This might explain the stronger effects of Regorafenib observed in vitro [12, 30].

While we found a pronounced effect on proliferation in vitro, Regorafenib unexpectedly enhanced early tumor development in vivo, increasing both the tumor area and the proliferation rate significantly. In contrast to these findings during early tumor development, the paradoxical, tumor-promoting effect of Regorafenib was lost at later stages with no significant differences in tumor size remaining between Regorafenib-treated and control animals. Given the defined endpoint of our animal study, we could not assess the impact on survival. However, Regorafenib led to an increase in the proportion of highly proliferating tumors with a Ki-67 index higher 55% both during early and later tumor development, suggesting a potential survival disadvantage following Regorafenib treatment at least in this subset of tumors. Furthermore, the fact that the tumor-promoting effect was most pronounced during early tumor development indicates the presence of primary rather than secondary resistance mechanisms to Regorafenib in the genetic mouse model.

Hypervascular tumors such as PNETs generally are highly dependent on the nutrient supply through neoangiogenesis and therefore represent promising candidates for anti-angiogenic therapy strategies. Several angiogenesis-targeting multikinase inhibitors other than Regorafenib have shown promising effects on tumor growth with prolonged survival rates in the RIP1Tag2-mouse model [31, 32]. There are various studies in the literature demonstrating an impact of Regorafenib also on endothelial cells in vitro and in vivo. For example, Regorafenib inhibits the proliferation of VEGF- or FGF2-stimulated human umbilical vascular endothelial cells (HUVECs), and exhibits significant inhibitory effects on growth-factor-mediated VEGFR2 and VEGFR3 autophosphorylation and on intracellular VEGFR3 signaling in HUVECs [15].

To further address this discrepancy, we also investigated the effects of Regorafenib on microvessel density. As expected, Regorafenib induced a significant reduction of blood vessels in the pancreatic tissue surrounding the islet tumors. However, within the tumors we observed a surprising increase rather than decrease of microvessel density. Our data demonstrate that Regorafenib was not able to reverse the angiogenic switch during tumor development or to reduce existing tumor vascularization during tumor progression in the RIP1Tag2 model. These findings are in line with several reports in the literature describing a primary resistance to anti-angiogenic inhibitors as a common problem in cancer therapy. Proposed resistance mechanisms include enhanced lymphangiogenesis mainly through upregulation of VEGF-A and VEGF-C, as demonstrated during treatment with Sunitinib [33, 34]. Furthermore, the coverage with pericytes has been shown to protect blood vessels as potential resistance mechanism. According to this hypothesis, an alternating administration of a PDGFR inhibitor targeting pericytes and a VEGFR inhibitor could be a combination treatment strategy to overcome pericyte-associated resistance [32, 33, 35, 36]. Since the induction of hypoxia-inducible factor 1α (HIF1α) is a well-known resistance mechanism following anti-angiogenic therapies, we examined its expression levels after Regorafenib treatment. However, we were not able to detect a relevant expression of HIF1α in the protein lysates of our neuroendocrine cells exposed to Regorafenib. Therefore, we conclude that upregulation of HIF1α as a potential escape mechanism mediating antiangiogenic drug resistance has no relevant impact in neuroendocrine tumors of RIP1Tag2 mice.

In our study, circulating serum levels of VEGF-A in Regorafenib-treated RIP1Tag2 mice did not correlate with the tumor vascularization. During early tumor development, a non-significant reduction of circulating VEGF-A could be observed upon Regorafenib, during late tumor progression a significant increase following Regorafenib treatment was observed. This increase in circulating VEGF levels could represent a compensatory resistance mechanism, as described in several clinical trials using anti-angiogenic targeted therapies including the multi-kinase inhibitor Sunitinib [37, 38]. Different mechanisms for this upregulation have been described, including the release of VEGF from the blocked receptor, VEGF secretion from internal stores or its compensatory upregulation due to hypoxia. The latter is unlikely to play a significant role in this study because blood vessels were not reduced and histological sections showed no signs of apoptosis and/or necrosis in the tumor. Interestingly, an increase of plasma VEGF has also been detected in non-tumor bearing mice after anti-angiogenic therapy, indicating a tumor-independent mechanism [39].

The tumor-promoting potential of the TME and its therapeutic targeting have already been extensively described in literature and are currently the focus of many clinical studies. Stroma-mediated effects include increased angiogenesis, enhanced tumor invasion and inhibition of anti-tumoral immune response.

Tumor-associated macrophages represent an important cellular component of the tumor stroma and are known as important mediators of tumor angiogenesis. As described in numerous other cancer entities, a correlation between macrophage infiltration, increased invasion and metastasis as well as worsened prognosis has been described in PNET [5–7, 40]. In this context, the macrophage growth factor CSF-1 has been identified as important driver of tumor growth and invasiveness [41–43]. Its absence has already been shown to reduce tumor growth in RIP1Tag2 mice, most likely due to inhibition of the angiogenic switch and subsequent tumor development [5].

To address potential non-tumor cell autonomous effects of Regorafenib, we also examined its impact on tumor-associated macrophages, in particular since CSF1R has been reported as target of Regorafenib [17]. Our in vitro experiments verified a time- and dose-dependent effect of Regorafenib on bone marrow-derived macrophages (BMM) of non-transgenic mice. Interestingly, after ex vivo polarization towards M1- or M2-macrophages, both M1- and M2-polarized macrophages showed higher viability and were less susceptible to Regorafenib than unpolarized M0 macrophages. CSF1R protein expression was very low in M1 macrophages compared to M0 and M2 macrophages, which is consistent with current literature indicating that CSF1R expression correlates to the M2 phenotype [18]. Interestingly, Regorafenib was able to reduce CSF1R protein expression in M0 but not in M2 macrophages. However, phosphorylation of CSF1R as indicator of its activation was affected by Regorafenib in both M0 and M2 macrophages. The low expression levels of CSF1R in M1 macrophages explains the lack of efficiency of Regorafenib in these cells in vitro. To date, it remains to be elucidated why M2 macrophages do not respond to Regorafenib despite responding with a reduced CSF1R activation.

In vivo, macrophages migrate into the tumor. There they acquire different phenotypes depending on the predominant microenvironmental cues. Although Regorafenib exerts strong effects on M0 macrophages in vitro, the relevance of these effects under physiological tumor conditions in vivo remains unclear. F4/80 positive macrophages were effectively reduced by Regorafenib in the surrounding normal pancreatic tissue. However, within the tumors the number of F4/80 positive macrophages was unexpectedly increased. Likewise, the proportion of CSF1R positive cells was increased under Regorafenib therapy. These results indicate that Regorafenib promotes the infiltration of macrophages into the tumor and these macrophages most likely exert tumor-promoting properties through the expression of CSF1R.

The reasons for this paradoxical attraction of macrophages to the tumor compartment, but not in the surrounding tissue remain to be elucidated. Since Regorafenib did not induce apoptosis in any of the macrophage subtypes in our in vitro studies, it is unlikely that the observed increase in M2 macrophages is due to a Regorafenib-induced apoptosis of M1 macrophages. Investigations with a comprehensive space- and time-resolved ex vivo characterization of the polarization patterns of tumor-infiltrating macrophages in the presence or absence of Regorafenib would be necessary.

Our in vivo data stand in contrast to our expectations and to published effects of Regorafenib in xenograft mouse models in the literature [15, 44]. An important and probably decisive difference between the published data and our study is the mouse model used for in vivo validation. We used a transgenic immunocompetent mouse model in which tumors develop gradually over time and are surrounded by a complex microenvironment. This is in contrast to xenograft models in athymic mice in which most of the previous in vivo data have been generated. It is conceivable that xenograft models lack compensatory resistance mechanisms which depend on an intact immune system.

In addition, the impact of tumor-associated macrophages appears to vary between tumor entities. Whereas tumor-associated macrophages in most cancers are associated with poor prognosis and increased invasiveness, in other tumors such as colorectal cancer they have been associated with a better outcome [45–47]. It can be speculated that the in vivo effects seen in our model are also dependent on a highly complex and tumor-(sub)type specific interaction with the surrounding micromilieu. In the stroma of the RIP1Tag2 mouse model, Regorafenib clearly induces a tumor-promoting immune response.

As one putative resistance mechanism occurring in vivo, we identified the upregulation of the pro-survival protein Bcl-2 which was upregulated upon Regorafenib exposure during the phase of early tumor development.

The targeted pharmacological inhibition of the survival-promoting Bcl-2 proteins by so-called “BH3 mimetics” therefore would represent a promising approach in the treatment of tumors. In solid tumors, such as breast and lung tumors, as well as various hematological diseases, the efficacy of BH3 mimetics on tumor growth has already been demonstrated [48, 49]. A combinatorial effect of ABT-263, an inhibitor of Bcl-2, Bcl-XL, and Bcl-w, together with anti-angiogenic TKI’s was already reported in HepG2 liver carcinoma cell lines in vitro. The BH3-mimetic potentiated the effect of sorafenib, which is structurally similar to Regorafenib, in HepG2 liver carcinoma cells in vitro and induced apoptosis by a caspase-dependent mechanism. In addition, a synergistic effect of sorafenib and ABT-263 in a subcutaneous HepG2 mouse model has been demonstrated [50].

Currently, there are no data on the role of Bcl-2 inhibitors in pancreatic neuroendocrine tumors. Likewise, studies on potential upregulation of Bcl-2 as resistance mechanism to the approved anti-angiogenic TKI sunitinib in PNET are lacking. According to our data it may be speculated that combined treatment strategies with anti-angiogenic TKI´s such as Regorafenib and BH3 mimetics in PNET’s could be able to overcome resistance to anti-angiogenic monotherapies in vivo.

Accumulating evidence highlights the importance of the TME including infiltrating immune cells in promoting tumor growth, invasion and metastasis [19]. The infiltrating immune cells often exert tumor-modulating effects during all stages of tumor progression that can vary greatly depending on the subtype, tumor entity and patient characteristics.

In addition to the tumor cell-autonomous Bcl-2-dependent resistance to Regorafenib, we identified a non-autonomous mechanism dependent on infiltrating macrophages that involves the COX2-PGE2 signaling reducing the susceptibility to regorafenib and promotes further tumor growth. COX2 (cyclooxygenase-2) is overexpressed in most solid tumors such as colorectal, liver, pancreatic, breast as well as lung cancer and its activity correlate with angiogenesis, invasion and resistance to chemotherapy. COX2-knock out mice showed an enhanced T cell survival and immune surveillance as well as a disrupted TAM-function. The enzymatic product of COX2 from arachidonic acid, PGE2, activates the prostaglandin E2 receptor 1–4 (EPs 1–4)-dependent signaling pathways mediating proliferation, survival, angiogenesis, migration and invasion [21–24].

In line with these data, we could show that in bone BMM COX2 and its enzymatic product PGE2 were predominantly increased in tumor-promoting M2, but also M0 macrophages after Regorafenib treatment in vitro.

Furthermore, the addition of PGE2 to murine pancreatic β-cells (HMEG) rescued the cytotoxic in vitro effect of Regorafenib to a significant extent. These data strongly suggest that PGE2, induced by Regorafenib in a non-tumor cell autonomous manner, adds to the resistance of the tumor cells detected in vivo.

However, the PGE2 addition to Regorafenib treated HMEG cells could not further increase the Bcl-2 expression in tumor cells, indicating that the Regorafenib-induced Bcl-2-upregulation seen in the tumor cells represents a COX2-PGE2 independent tumor cell-autonomous resistance mechanism.

## Conclusions

The results of this study reinforce the growing body of evidence that the in vitro effects of anti-angiogenic multi-kinase inhibitors are frequently counteracted by complex resistance mechanisms in vivo which may at least in part mediated by cues from the inflammatory stroma which are still poorly understood. In a preclinical setting it is essential to characterize the in vivo effects in immunocompetent genetic mouse models with a stroma reaction recapitulating human disease. To overcome in vivo resistance mechanisms, combinatorial strategies including simultaneous targeting of survival pathways such as Bcl-2 or immunomodulatory approaches such as CSF1R targeting have to be considered to achieve a sustained therapeutic efficacy in vivo.

### Supplementary information

Supplemental material

Arrive Compliance Questionnaire

## Data Availability

All data generated or analyzed during this study are included in this published article and its supplementary information files.
