# Supplementary material for: Anti-angiogenic therapy using the multi-tyrosine kinase inhibitor Regorafenib enhances tumor progression in a transgenic mouse model of ß-cell carcinogenesis
Source: Br J Cancer. 2023 Aug 24;129(8):1225–37. doi: 10.1038/s41416-023-02389-6 (PMC10575939; doi:10.1038/s41416-023-02389-6)
Supplement: Supplementary file 1 — Supplemental material [file 41416_2023_2389_MOESM1_ESM.docx]

**Anti-angiogenic therapy using the multi-tyrosine kinase inhibitor Regorafenib enhances tumor progression in a transgenic mouse model of ß-cell carcinogenesis**

model of ß-cell carcinogenesis

**Maren Juliane Egidi ^1^, Sebastian Krug ^1^, Johannes Haybaeck ^2 3^, Patrick Michl* ^1 4^ and Heidi Griesmann* ^1^**

**Supplementary material – tables and figures**

**Supplementary table 1: primer sequences**

| **name** | **Forward primer** | **Reverse primer** |
| --- | --- | --- |
| CCL5 | TCCCTGTCATTGCTTGCTTGCTCTAG | GAGCAGCTGAGATGCCCATT |
| COX2 | TTGATATGTCTTCCAGCCCATTG | ACGGAACTAAGAGGAGCAGCAA |
| Dectin | GGAATCCTGTGCTTTGTGGTAGTAG | GGAAGGCAAGA5‘CTGAGAAAAACCTC |
| iNOS | CCAAGCCCTCACCTACTTCC | CTCTGAGGGCTGACACAAGG |
| Insulin | CACAACTGGAGCTGGGTGGA | TAGGCTGGGTAGTGGTGGGT |
| Mgl2 | TTAGCCAATGTGCTTAGCTGG | GGCCTCCAATTCTTGAAACCT |
| PDX1 | GAGAGCCAGTTGGGTATAG | ATTGGTCCCAGGAAAGAG |
| RIP1Tag2 | GGACAAACCACAACTAGAATGCA | CAGAGCAGAATTGTGGAGTGG |


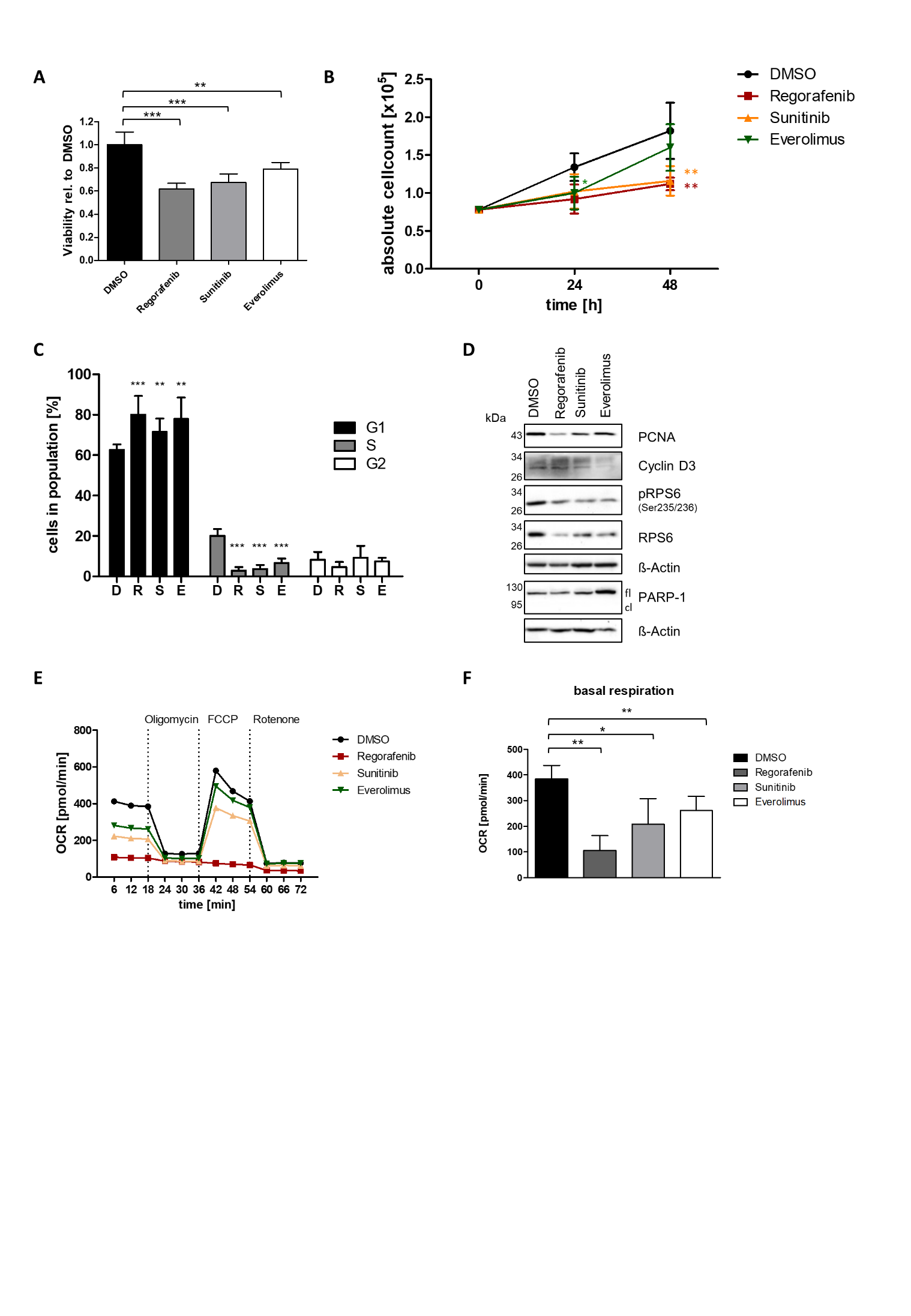


**Supplementary Figure S1:** Regorafenib reduces cell proliferation and viability more effectively than Sunitinib und Everolimus in QGP-1 cell line. QGP-1 cells were treated with 6µM Regorafenib, 10µM Sunitinib, 1uM Everolimus or 0.1% DMSO for 24h and 48h respectively. **(A)** Effect on cell viability after 48h as assessed by an ATP-based CellTiter Glo Assay. Values are shown relative to DMSO-control. **(B)** Cell proliferation shown as absolute cell counts after 24h and 48h. Statistical significance of each treatment compared to control at 24h and 48h, respectively. **(C)** Cell cycle analysis was done by flow cytometry using Propidium iodide staining. Figure shows percentage of cells in G1-, S- and G2-phase after 48h treatment with DMSO (D), Regorafenib (R), Sunitinib (S) and Everolimus (E) respectively. The decrease of the S-phase was significant after Regorafenib, Sunitinib and Everolimus treatment with p-values of 0.0001, 0.0002 and 0.0005, respectively. **(D)** Representative Western blot analysis showing expression of proliferation and translation markers as well as apoptotic protein PARP-1 (fl: full-length PARP-1; cl:cleaved PARP-1 fragment at 89 kDa). β-actin was used as loading control. **(E)** Mitochondrial respiration after 24h treatment depicted in a temporal course **(F)** and bar chart **(G)**. Oxygen consumption rate (OCR) in pmol/min. Recordings took place under basal, oligomycin-inhibited, FCCP-induced maximal and rotenone + antimycin A-inhibited (rotenone) conditions. Experimental data are representative for at least three independent experiments and are presented as means ± SD. Statistical evaluation was performed by the use of a two-tailed unpaired Student´s t test. *p≤0.05; **p<0.01; ***p<0.001.

**Supplementary Figure S2:** Regorafenib reduces cell proliferation and viability more effectively than Sunitinib und Everolimus in BON-1 and QGP-1 cell line. **(A)** BON-1 and **(B)** QGP-1 cells were treated with Regorafenib (4µM, 6µM), Sunitinib (5µM, 10µM), Everolimus (0.5, 1µM) or 0.1% DMSO for 24h and 48h respectively. Time- and dose-dependent effect on cell viability after 24h and 48h was performed by an ATP-based CellTiter Glo Assay. Values are shown relative to DMSO-control. Data are representative for at least three independent experiments and are presented as means ± SD. Statistical evaluation was performed by the use of a two-tailed unpaired Student´s t test. *p≤0.05; **p<0.01; ***p<0.001; ns not significant.

**Supplementary Figure S3:** Effect of Regorafenib, Sunitinib und Everolimus on the cell metabolism in BON-1 and QGP-1 cell line. **(A)** BON-1 and **(B)** QGP-1 cells were treated with 6µM Regorafenib, 10µM Sunitinib, 1uM Everolimus or 0.1% DMSO for 1h. Mitochondrial respiration was assessed after 1h treatment. Oxygen consumption rate (OCR) in pmol/min. Recordings took place under basal, oligomycin-inhibited, FCCP-induced maximal and rotenone + antimycin A-inhibited (rotenone) conditions. **(C)** BON-1 and **(D)** QGP-1 cells were treated with 6µM Regorafenib, 10µM Sunitinib, 1uM Everolimus or 0.1% DMSO for 24h. Extracellular acidification rate (ECAR) in mpH/min. Recordings took place under basal, oligomycin-inhibited, FCCP-induced maximal and rotenone + antimycin A-inhibited (rotenone) conditions.

**Supplementary Figure S4:** Characterization of murine pancreatic β-tumor cell lines (HMEG**).** HMEG 1-3 isolated from 15-week-old RIP1Tag2-mice were confirmed as pancreatic neuroendocrine cells by **(A)** Western blot analysis determining the expression of Chromogranin A and SV-40. *β*-actin was used as a loading control. **(B)** The amount of insulin and PDX-1 on RNA level was evaluated by qRT-PCR and normalised to RPLP0.

**Supplementary Figure S5:** Regorafenib reduces cell viability and cell proliferation in murine pancreatic β-tumor cell lines (HMEG). HMEG cells were treated with increasing Regorafenib concentrations ranging from 4 µM – 10µM or DMSO (0.1%) for 24h and 48h, respectively. **(A)** Effect on cell viability was assessed by an ATP-based CellTiter Glo Assay. Values are shown relative to DMSO-control. Data are representative for at least three independent experiments and are presented as means ± SD. HMEG cells were treated with 10µM Regorafenib or DMSO (0.1%) for 48h. **(B)** The amount of proliferating (EdU-positive) cells is displayed in a histogram as an overlay based on the measured Alexa 488 fluorescence signals by flow cytometry analysis. **(C)** Percentage of proliferating (EdU-positive) cells is displayed based on the measured Alexa 488 fluorescence signals by flow cytometry analysis (n=1).


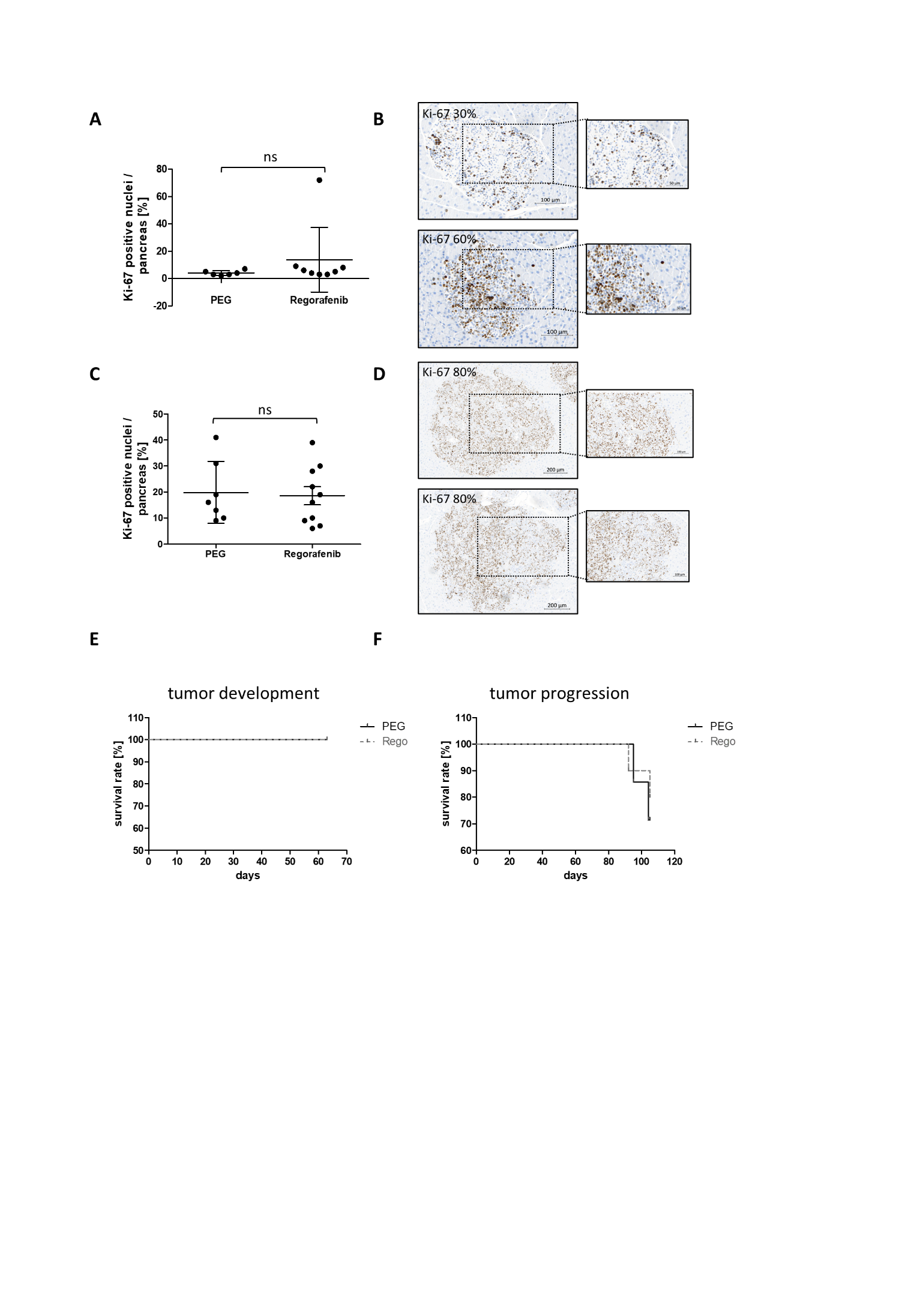


**Supplementary Figure S6:** Regorafenib leads to a higher proliferation rate during tumor development but shows no effect during late tumor progression. Percentage of Ki-67-positive nuclei in the entire pancreatic tissue during early tumor development **(A)** and late tumor progression **(C)** of Regorafenib- and PEG-treated mice with representative Ki-67 stainings of individual exemplary islets **(B+D)**. Box plot data are presented as mean±SD by Mann–Whitney U test; ns=not significant. **(E)** Survival analysis according to Kaplan-Meier during tumor development. In this study, 6 animals were treated with PEG control solution and 8 animals with Regorafenib from week 6-9. **(F)** Survival analysis according to Kaplan-Meier during tumor progression. In this study, 7 animals were treated with PEG control solution and 10 animals with Regorafenib from week 9 -15. The y-axis describes the proportion of living mice in a treatment group in a linear percentage scale. The x-axis indicates the survival time in days. No significant difference was evaluated by log-rank test in both trials (p>0.05).


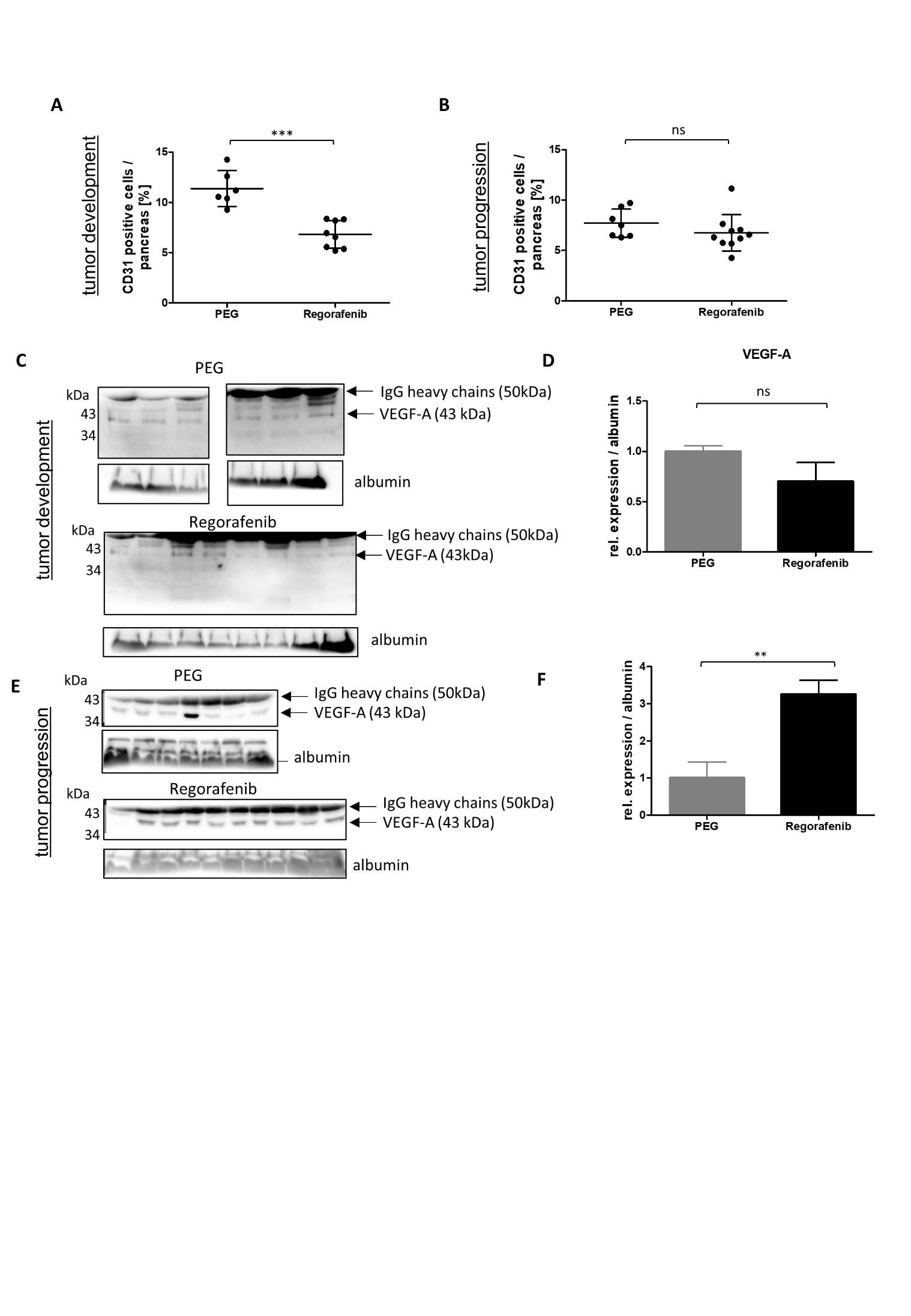


**Supplementary Figure S7:** Quantification of CD31-positive cells. Percentage of CD31-positive cells in the entire pancreatic tissue during tumor development **(A)** and tumor progression **(B)** of Regorafenib- and PEG-treated mice. Boxplot data are presented as mean±SD by Mann–Whitney U test. ***p<0.001; ns=non-significant. Western blot analysis with statistical analysis of VEGF-A in the sera of PEG- and Regorafenib-treated mice during tumor development **(C+D)** and tumor progression **(E+F)**. Albumin was used as control. All samples were run on one single gel, but additional treatment conditions that were originally loaded on the gel were cut out.

**Supplementary Figure S8:** M1 and M2 markers in polarized macrophages. The polarization of primary murine macrophages towards a M1 (by LPS+IFNy) **(A)** and a M2 phenotype (by IL-4) **(B)** was confirmed by the expression of characteristic M1 markers iNOS (*Inducible nitric oxide synthase*) and CCL5 as well as M2 markers Mgl2 (*macrophage galactose-type C-type lectin 2*) and Dectin on RNA level shown by representative qRT-PCR-analysis, normalised to RPLP0.


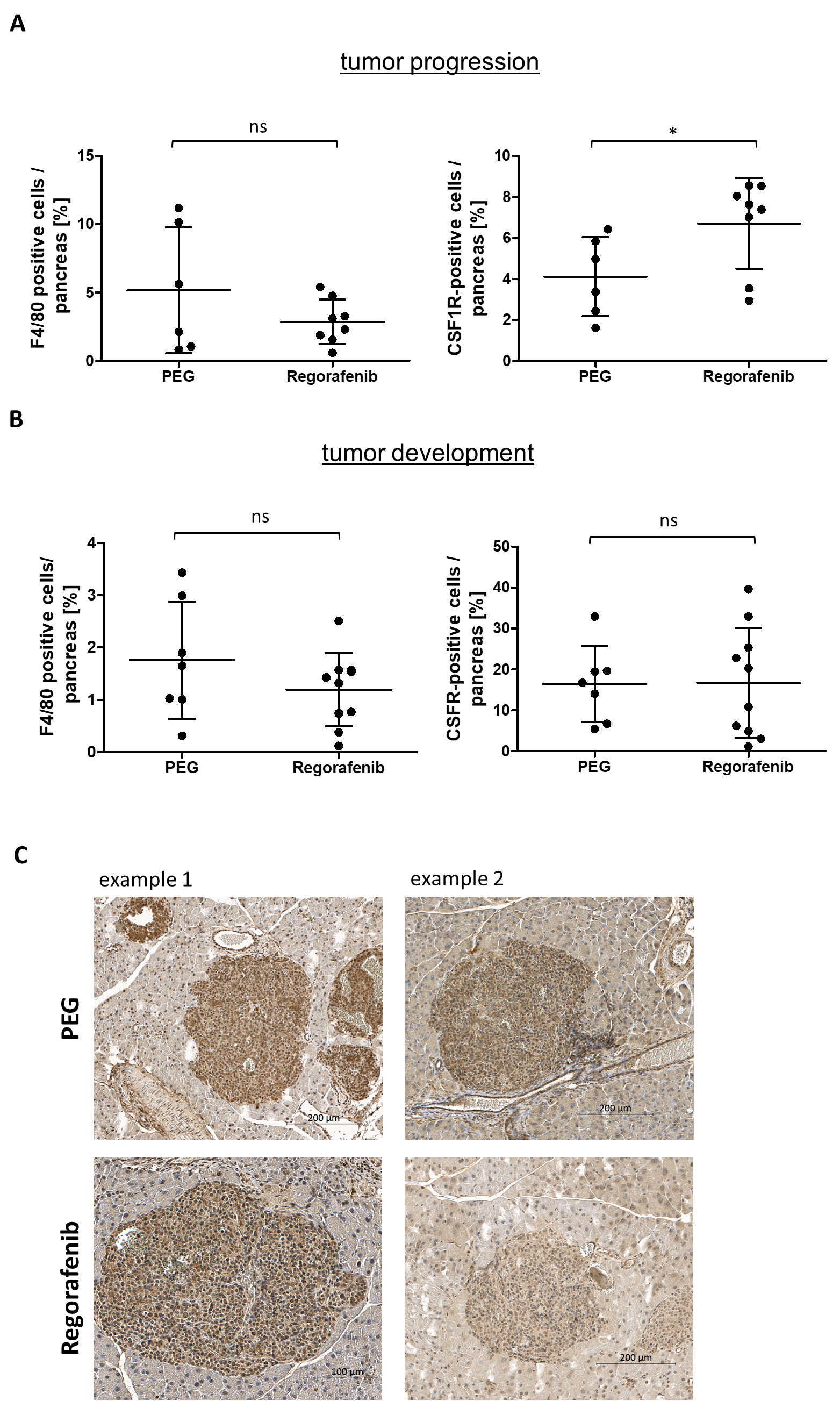


**Supplementary Figure S9:** Quantification of F4/80 and CSF1R-positive cells. Percentage of F4/80- and CSF1R-positive cells in the entire pancreatic tissue during tumor development **(A)** and tumor progression **(B)** of Regorafenib- and PEG-treated mice. Boxplot data are presented as mean±SD by Mann–Whitney U test. *p≤0.05; ns=non-significant. **(C)** Representative CD3-staining of two each PEG- and Regorafenib-treated mice during tumor development.

**
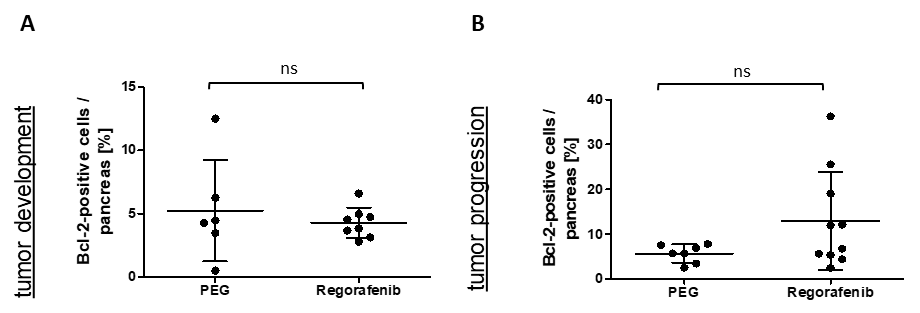
**

**Supplementary Figure S10:** Quantification of pro-survival Bcl-2-protein *in* ***vivo*.** Percentage of Bcl-2-positive cells in the entire pancreatic tissue during tumor development **(A)** and tumor progression **(B)** of Regorafenib- and PEG-treated mice. Boxplot data are presented as mean±SD by Mann–Whitney U test. ns=non-significant.
